# From Degeneration to Regeneration: The Evolving Landscape of Cell-Based Tendon Repair

**DOI:** 10.3390/cells15161483

**Published:** 2026-08-18

**Authors:** Ines Wang, Brett D. Owens, Jay Trivedi

**Affiliations:** Department of Orthopedics, Alpert Medical School of Brown University, Rhode Island Hospital, Providence, RI 02903, USA

**Keywords:** tendon repair, regenerative medicine, cell-based therapy, mesenchymal stem cells, tenogenic differentiation, extracellular vesicles, tendinopathy, paracrine signaling, extracellular matrix remodeling

## Abstract

**Highlights:**

**What are the main findings?**

- Distinct stem cell populations demonstrate differential regenerative potential in tendon repair, with source-dependent variation in tenogenic commitment, hypoxic tolerance, and immunomodulatory capacity emerging as key determinants of therapeutic outcome.
- Cell-based tendon repair operates through mechanistically distinct paracrine axes, including angiogenesis, collagen remodeling, immunomodulation, and tenogenic differentiation, each governed by discrete and targetable signaling cascades.
- Limited cell survival, absence of standardized dosing protocols, and lack of large-animal and phase II trial data remain the critical barriers to clinical translation.

**What are the implications of the main findings?**

- Emerging clinical trial evidence supports the therapeutic potential of cell-based approaches in tendon repair, with outcomes influenced by patient selection, delivery parameters, and post-operative rehabilitation protocols.
- Patient-specific cell selection (stratified by injury type, anatomical site, age, and metabolic status), and autologous vs. allogeneic selection, represent the defining challenge and opportunity for next-generation tendon regenerative strategies.

**Abstract:**

Tendinopathies represent a major clinical challenge. Vasculature, neuromuscular junctions, low cellularity, and slow extracellular matrix (ECM) turnover restrict endogenous repair and predispose injured tendons to fibrosis, mechanical weakness, and reinjury. Current therapeutic strategies including rehabilitation protocols, anti-inflammatory medications, platelet-rich plasma (PRP) injections, and surgical repair primarily address symptoms or structural deficits without correcting the underlying biological limitations of tendon healing. Cell-based therapies have emerged as a promising regenerative approach aimed at restoring tissue homeostasis through modulation of angiogenesis, collagen synthesis, immune responses, and tenogenic differentiation. Mesenchymal stem cells (MSCs), adipose-derived stem cells (ADSCs), tendon-derived stem cells (TDSCs), induced pluripotent stem cells (iPSCs), differentiated tenocytes, and extracellular vesicle (EV)-based products have demonstrated the ability to enhance vascularization, promote type I collagen remodeling, suppress excessive inflammation, and stimulate tenocyte lineage commitment. These effects are mediated through paracrine signaling, growth factor secretion, and activation of key pathways, including HIF-1α, TGF-β/SMAD, NF-κB, and PI3K/Akt signaling. Despite promising preclinical data, significant translational challenges remain, including limited cell survival at the injury site, variability in cell sources and dosing, immunogenicity, risk of misdifferentiation, and lack of standardization across clinical protocols. Emerging strategies such as genetic modification, hypoxic preconditioning, scaffold-based delivery systems, and extracellular vesicle engineering aim to enhance therapeutic efficacy and reproducibility. This review synthesizes current evidence on cell-based tendon repair, critically evaluates mechanistic insights, clinical trials, and translational barriers, and outlines future directions toward biologically informed regenerative therapies.

## 1. Background

Tendons are dense, fibrous connective tissues that serve as the mechanical link between muscle and bone, enabling efficient force transmission and coordinated movement. Their morphology and the hierarchical organization and composition of their extracellular matrix (ECM) provide them with high tensile strength and viscoelasticity, allowing tendons to function effectively with relatively low metabolic activity [1,2,3]. This complex architecture allows them to withstand substantial tensile loads during low-impact daily activity as well as high-impact physical activity. Despite this mechanical specialization, tendons exhibit limited intrinsic regenerative capacity, making them particularly vulnerable to chronic dysfunction following injury [4,5]. While critical to maintain tendon homeostasis under normal conditions, the dense ECM network and limited availability of resident cells hinder the delivery of reparative cells, oxygen, and nutrients in pathological states [6,7]. Consequently, tendon healing is often incomplete, leaving patients susceptible to scarring and/or reinjury [8].

Similarly, tendons facilitate collagen turnover and tissue remodeling with a relatively low ECM synthesis rate compared to other connective tissues [9]. Gradually increasing the mechanical load can enhance these turnover rates, adaptively reducing tissue stress and improving load resistance [10,11]. However, repetitive sudden or excessive loading can disrupt this balance, leading to a state in which ECM synthesis cannot keep up with matrix degradation. Such overload places tendons at risk of acute tendonitis and in the long term, with persistent load and aberrant healing, chronic degenerative tendinopathy. Chronic degenerative tendinopathy is characterized by progressive collagen disorganization, increased proteolytic activity, tenocyte apoptosis, and impaired healing capacity, which ultimately results in pain, stiffness, and reduced mechanical resilience [12]. Current strategies fall short at lessening these symptoms as they fail to directly address the intrinsic physiological limitations, underlining a need for novel cell-based approaches [13]. Despite decades of surgical and biological innovation, no current treatment reliably restores the native mechanical integrity, cellular density, and organized extracellular matrix architecture of injured tendon tissue. This represents a fundamental unmet clinical need, as a substantial proportion of patients experience persistent pain, functional deficits, and unacceptably high rates of reinjury.

The field of cell-based tendon repair has advanced considerably, yet a unified framework bridging mechanistic biology, comparative cell analysis, and clinical translation remains absent. This comprehensive review addresses that critical gap. We provide a detailed head-to-head comparative evaluation of commonly studied stem/progenitor cell populations. Additionally, we examine and comprehend not only their regenerative capacity but also their translational readiness, limitations, and clinical trial evidence. The goal of this review is to provide a decision-making resource for researchers and clinicians, one that moves beyond cataloging what each platform does, toward defining how, why, and for whom each strategy is most likely to succeed.

## 2. Current Strategies for Tendon Repair

For minor tendinitis or tendinopathy, conservative approaches are preferred and include rest, activity modification, physical therapy (PT), rehabilitative exercise, and non-steroidal anti-inflammatory drugs (NSAIDs) [14]. More invasive strategies involve corticosteroid (CS) injections, platelet-rich plasma (PRP) injections, and acupuncture, while severe cases require surgical repair. These strategies are typically used in tandem to provide growth and healing factors, minimize inflammation and pain, and rebuild tendon strength. Their efficacy, however, remains highly inconsistent.

Conservative management strategies such as activity modification, PT, and structured rehabilitation primarily focus on functional improvement. Appropriately dosed rest, load, or exercise may stimulate gradual remodeling of collagen fibers and improve tensile strength via mechanotransduction [15,16]. However, this process is slow and does not fully reverse the degenerative changes in tendon tissue. Instead, it enhances tendon tolerance and structural organization rather than restoring normal native tendon architecture [17].

NSAIDs are typically used for alleviating pain and swelling associated with inflammation and may provide short-term symptom relief, allowing patients to better tolerate loading and rehabilitation [18]. However, tendinopathies are now widely understood to be mostly degenerative rather than inflammatory in nature, limiting the extent to which NSAIDs address the underlying pathology [19]. In addition, they are not suitable for long-term use, as chronic administration is associated with adverse gastrointestinal, cardiovascular, renal, cerebrovascular, and central nervous system effects [20,21]. Similarly, CS injections are administered for their potent anti-inflammatory properties that offer rapid short-term pain relief. However, these benefits are often transient, and repeated use may actually impair tendon healing by reducing collagen synthesis, tenocyte activity, and matrix repair, which may further increase the risk of tendon degeneration and rupture [22].

Surgical intervention is typically reserved for severe or chronic cases, particularly when conservative management fails or when partial or complete tendon rupture is present. Procedures vary depending on severity and location of injury, and may involve tissue debridement, arthroscopic techniques, tendon release, and reconstructive repair [19]. They may restore tendon continuity and improve biomechanical function; however, these procedures do not fully address the biological deficits of tendon degeneration and may therefore result in retearing or other complications. Notably, the reported retear rate for the arthroscopic repair of traumatic full-thickness rotator cuff tears between 2014 and 2021 was 27.3% [23]. Of those who were surgically treated for flexor tendon injuries, 17% reported major complications, including rerupture, adhesion, and infection [24].

Platelet-rich plasma (PRP) is an autologous blood-derived concentrate containing a supraphysiological abundance of platelets and associated growth factors, including PDGF, TGF-β, VEGF, and IGF, which collectively promote tenocyte proliferation, collagen synthesis, angiogenesis, and inflammation modulation [25,26,27,28,29,30,31]. PRP is widely used as a therapeutic approach for soft-tissue repair, including tendons. Its clinical appeal lies in its autologous nature, point-of-care preparation, and ability to recapitulate aspects of the early healing cascade that tendon tissue inherently lacks. As a result, PRP has been widely investigated as an adjunct to both conservative and surgical tendon repair across multiple anatomical sites [32,33,34,35]. A 2025 meta-analysis of 13 studies with 697 patients with chronic Achilles tendinopathy (CAT) found that patients treated with PRP experienced reductions in pain and improvements in function, with many returning to previous levels of activity [36]. Contrastingly, a 2025 systematic umbrella review reported no significant differences between PRP and saline control groups for CAT [37]. While PRP treatment has shown promising results for tendon repairs in preclinical and clinical studies, there are factors associated with PRP treatment that have variable clinical outcomes [37,38].

These inconsistent outcomes attributed to PRP largely reflect formulation heterogeneity rather than a fundamental biological limitation [39,40,41]. PRP preparations differ substantially in platelet concentration, leukocyte content, activator type, and preparation protocol, variables that produce distinct growth factor profiles and divergent cellular responses in the tendon microenvironment [42,43]. Leukocyte content is a particularly critical and underreported determinant of outcome: leukocyte-rich PRP retains neutrophils and monocytes that amplify pro-inflammatory cytokines including IL-1β and TNF-α, which may be detrimental to tendon homeostasis, whereas leukocyte-poor PRP may be preferable in degenerative conditions where chronic inflammation is already a primary pathological driver [44]. Activator type further governs efficacy through growth factor release kinetics, as thrombin induces rapid platelet degranulation while calcium chloride promotes slower and more sustained growth factor release—a distinction with direct implications for therapeutic duration and tissue response [45]. Additionally, a large number of published PRP studies do not report leukocyte concentrations, precluding meaningful cross-study comparison and accounting for much of the apparent contradiction in the literature [32].

Although its therapeutic benefit remains uncertain, autologous PRP continues to be used in tendon repair due to its excellent safety profile and lack of immunogenic or tumorigenic effects. Future trials should adopt standardized reporting frameworks such as the PAW classification to enable rigorous, formulation-stratified evaluation of this compositionally heterogeneous therapy.

## 3. Cell-Based Therapeutic Approaches

Cell-based therapy offers a promising, less contradictory alternative, supplying exogenous, most often progenitor, cell populations to compensate for these physiological limitations. In cases where tissue integrity is compromised, replacement of lost tissue is required via implantation with a matrix carrier. When tissue integrity remains intact, as in degenerative tendinopathy, cells may instead be administered via injection, either systemically or locally. Systemic delivery bears the risk of pulmonary entrapment during cell homing, making local injection the preferred approach [46]. Local administration enables the direct delivery of cells to the injury site, and is often performed in multiple rounds and in combination with mechanical stimulation and scaffolds (hydrogels, biomaterials, 3D scaffold) to promote adequate cell–cell interaction, integration, and retention [47,48]. Beyond delivery strategy and scaffold composition, additional parameters, such as cell type, cell quantity, and growth factor supplementation, must be optimized to improve therapeutic outcomes [49].

## 4. Cell Types and Sources

Cells for cell-based therapies can be autologous or allogenic, derived from the patient or a donor, respectively. Autologous therapeutics represent a form of personalized medicine, carrying minimal risk of immune rejection or graft-versus-host responses since cells are genetically identical to the recipient [50]. However, this approach is only feasible if the patient possesses healthy cells that can be effectively isolated and expanded in vitro—a process that becomes increasingly challenging with age or pre-existing medical conditions. In contrast, allogeneic therapies, derived from healthy donors, offer greater potential for standardization and large-scale manufacturing, but carry a higher risk of immune incompatibility [50].

Cell types investigated for tendon repair range from totipotent and pluripotent cells, capable of differentiating into nearly any cell type, to multipotent mesenchymal stem cells (MSCs) and terminally differentiated cells. These populations are commonly classified by their source, and include fetal and embryonic cells (ESCs), amniotic- and placenta-derived cells, bone marrow-derived MSCs (BM-MSCs), adipose-derived stem cells (ADSCs), synovium-derived stem cells, tendon-derived stem cells (TDSCs), endothelial cells (ECs), induced pluripotent stem cells (iPSCs), and fully differentiated tenocytes. Various preclinical studies have demonstrated the efficacy of these cell types for tendon repair.

Among these, multipotent BM-MSCs and ADSCs are the most widely studied and have become the reference cell populations for stem cell-based approaches [51,52,53]. ADSCs are readily harvested through liposuction, yielding large cell numbers, whereas BM-MSCs are less plentiful and require a more invasive puncture. Although they possess lower pluripotency compared to ESCs, both cell sources circumvent many of the ethical and regulatory challenges associated with ESCs, making them particularly appealing for translational applications. iPSCs combine the advantages of both; they are fully pluripotent like ESCs yet free from the associated ethical concerns as they are rejuvenated or reprogrammed from adult somatic cells. However, as all progenitor cells have the potential to differentiate into chondrocytes, osteoblasts, and adipocytes, some researchers may instead opt for cells more inherently primed towards tenocyte differentiation, like TDSCs, or fully differentiated tenocytes [54]. A head-to-head comparison of six major cell types is summarized in Table 1.

### 4.1. Bone Marrow-Derived Mesenchymal Stem Cells (BM-MSCs)

BM-MSCs are the earliest and most extensively studied cell populations for tendon regeneration, although they are generally limited in abundance and require an invasive aspiration procedure for harvest [51,55]. These stromal stem cells can self-renew and differentiate into various cell lineages, primarily adipocytes, osteoblasts, and chondrocytes [55]. Additional lineage fates, including tenocyte-like phenotypes, can be induced under specific culture conditions and with mechanical or biochemical stimulation [55,56].

Numerous preclinical studies have demonstrated that BM-MSCs improve tendon healing by enhancing ECM remodeling and composition, collagen organization, and biomechanical strength. In an ovine Achilles collagenase-induced tendinitis model, Crovace et al. found that both cultured and mononuclear BM-MSCs restored native tendon composition relative to fibrin and saline controls [57]. Histological and immunohistochemical analyses showed that BM-MSC treatment significantly increased the expression of healthy tendon markers, including collagen type I and cartilage oligomeric matrix protein (COMP), while decreasing collagen type III expression [57]. Similarly, in an equine superficial digital flexor tendon (SDFT) injury model, Smith et al. demonstrated that a combination of BM-MSC injection and rehabilitation resulted in histological organization and tendon composition that more closely resembled native tendon compared with controls [58].

In terms of tendon fiber architecture, Yokoya et al. evaluated autologous BM-MSCs delivered on a polyglycolic acid (PGA) scaffold in a rabbit rotator cuff model versus scaffold-only controls [59]. The BM-MSC group exhibited organized fibrocartilage formation at the tendon belly and aligned Sharpey fiber insertion at the tendon–bone interface, while controls showed fibroblast disorganization throughout. Biomechanically, these structural improvements translated into significantly greater tensile strength at 16 weeks following implantation. Likewise, ex vivo studies have demonstrated functional benefits of BM-MSCs in flexor tendon repair models. In a canine lacerated flexor tendon model, collagen gel patches seeded with or without BM-MSCs were placed between the two severed tendon ends. BM-MSC-treated tendons showed increased failure strength and stiffness at 2 and 4 weeks, supporting their role in improving mechanical integrity in early-stage tendon repair [60].

Evidence also suggests that these benefits may extend to long-term functional outcomes. Autologous BM-MSCs were implanted into superficial digital flexor tendon (SDFT) lesions in 168 racehorses under ultrasonographic guidance, followed by a structured 48-week rehabilitation program [61]. At long-term follow-up, treated horses demonstrated a reinjury rate of 18%, compared with 56% in conventional management cases, suggesting that BM-MSC therapy in combination with rehabilitation may reduce the risk of recurrent injury.

In addition to the direct effects to the tendon tissue itself, BM-MSCs may also influence the surrounding musculoskeletal environment. Flück et al. demonstrated that intramuscular BM-MSC injection reduced muscle-to-fat-conversion of the infraspinatus muscle in an ovine RCT model, preserving muscle strength and function during tendon healing [62]. This study suggests that BM-MSC therapy may not only restore tendon composition and organization, but help maintain the essential mechanical interactions between, and properties of, tendons and neighboring muscles.

Additionally, stem cell lineage fates can be induced using various preconditioning methods. One strategy for enhancing the tenogenic potential of BM-MSCs involves targeted modulation of tendon-specific transcriptional programs. SCX, a key transcription factor involved in tendon cell lineage specification, represents a promising target for directing BM-MSCs toward a more tendon-like phenotypic fate. Gulotta et al. used adenoviral transduction to overexpress SCX in BM-MSCs for delivery in a rat rotator cuff model [63]. Rats who received SCX-BM-MSCs had enhanced TBI fibrocartilage formation and improved biomechanical properties, compared with unmodified BM-MSCs. With this, SCX overexpression may promote a more specialized tenogenic phenotype in BM-MSCs, and in turn enhance their structural and functional regenerative potential.

### 4.2. Adipose-Derived Stem Cells (ADSCs)

ADSCs have gained increasing attention due to their abundance, ease of isolation, and high proliferative capacity. Their accessibility and rapid expansion ability make them particularly suitable for translational and large-scale regenerative applications. Typically harvested through minimally invasive liposuction procedures, ADSCs can be obtained in substantially greater quantities than BM-MSCs [52]. Like BM-MSCs, they possess multilineage differentiation potential, although Dai et al. have reported that ADSCs exhibit slightly lower tenogenic differentiation capacity in vitro [64].

In a rat Achilles tendon model, ADSCs delivered within a hydrogel scaffold improved tendon healing, with enhanced collagen fiber alignment, increased tenogenic marker expression (SCX, TNMD, collagen I/III), and improved biomechanical function. These effects were further augmented when ADSCs were preconditioned with tenogenic growth factors, including growth differentiation factor-6 and platelet-derived growth factor-BB, prior to implantation. These groups also exhibited a lower collagen fiber dispersion range and mean histological scores, approaching those of native tendon [65]. de Lima Santos et al. assessed ADSC therapy from both gross morphological and biomechanical perspectives. In a rabbit flexor digitorum superficialis tendon injury model, ADSC injection combined with suture repair improved gross tendon morphology compared with suture-only controls, characterized by homogenous tissue appearance and reduced adherence to surrounding tissues. Biomechanically, ADSC-treated groups showed increased ultimate tensile load and deformation capacity, suggesting improved compliance during loading. However, an increase in cross-sectional area was observed, which may have implications for tendon gliding function, although its consequences were not directly assessed in this study [66].

As the choice of stem cell delivery method can influence therapeutic outcomes, strategies that improve cell retention and engraftment have received increasing attention. Shin et al. developed temperature-responsive cell sheets of ADSCs that preserve cell–cell contact while facilitating attachment to the tissue surface. In a chronic rat RCT model, these cell sheets were transplanted onto the tear site following surgical repair. Compared with repair-only and cell sheet-only groups, the ADSC sheet transplantation after repair group exhibited a larger fibrocartilage area, higher bone volume/total volume (BV/TV) values, and improved biomechanical scores [67].

These benefits may hold true in aged and estrogen-deficient (OVX) populations, as Veronesi et al. demonstrated that indirect co-culture of OVX tenocytes and ADSCs from both healthy young (sham) and OVX donors increased tenocyte proliferation, healing rate, and collagen I/III ratio, with more pronounced effects in sham-derived ADSCs [68]. However, Ahrberg et al.’s study in a large animal model has highlighted potential limitations in the translation of ADSC therapies. In their equine SDFT tendinopathy model, ADSC injection into the lesion only resulted in transient inflammation and a temporary increase in lesion size, with no major improvements in the immediate term. While ADSCs demonstrate promising regenerative effects in controlled small animal experimental models, their therapeutic efficacy may be influenced by culture conditions, injury type, delivery method, and species-specific responses [69].

### 4.3. Tendon-Derived Stem Cells (TDSCs)

TDSCs represent a resident stem/progenitor population isolated directly from tendon tissue, and are considered intrinsically primed toward tenogenic differentiation [70]. Compared with BM-MSCs, TDSCs exhibit higher basal expression of tendon-related markers, including SCX, TNMD, COMP, tenascin-C, and collagen I, reflecting their native tissue microenvironment [71,72]. This lineage predisposition reduces the need for extensive induction protocols and may enhance their suitability for tendon-specific regenerative applications, especially with exposure to tendon ECM-specific growth factors or environmental cues [72].

Following transplantation into ruptured rat Achilles tendons, TDSC treatment resulted in superior healing relative to BM-MSCs, evidenced by denser, more organized collagen architecture, increased expression of tendon-related ECM genes (tenascin-C, collagen I/III), greater early biomechanical strength, and higher ultimate failure load [73]. Although BM-MSC-mediated repair also improved tendon healing, TDSCs promoted more effective structure and functional restoration, likely reflecting their tendon-specific regenerative phenotype. Non-comparative in vivo studies similarly support the regenerative capacity of TDSCs in promoting tendon repair [74].

These regenerative effects can be further enhanced by tenogenic preconditioning strategies, including connective tissue growth factor (CTGF), ascorbic acid treatment, and SCX overexpression. CTGF supports cell adhesion, wound repair, and ECM remodeling, while ascorbic acid is essential for collagen synthesis and maturation. In a rat patellar tendon window injury study, CTGF- and ascorbic acid-preconditioned TDSCs produced thicker, better-aligned collagen fibrils and improved biomechanical properties following transplantation [75]. Similarly, lentiviral transduction of SCX into TDSCs accelerated early-stage tendon regeneration, highlighting the importance of signaling pathways in optimizing TDSC-mediated repair [76].

The incorporation of biomaterial scaffolds provides structural support for cell attachment, proliferation, and delivery, while also supplying biochemical cues for tissue repair. Wen et al. seeded TDSCs onto acellular porcine Achilles tendon (APAT) patches for rabbit rotator cuff repair, and observed that TDSCs grew even faster in the APATs than on conventional plastic surfaces [77]. Compared with rabbits treated with unseeded APATS, those receiving TDSC-seeded APATs exhibited improved organization of tendon–fibrocartilage–bone structure and biomechanical properties. Geng et al. further enhanced this strategy by integrating biochemical cues into a TDSC-seeded triphasic silk-fibrin (SF) scaffold, composed of distinct collagen I, TGF-B1, and hydroxyapatite (HA) phases that align with the hierarchical structure of the tendon–bone junction (TBJ) [78]. These phase-specific biochemical cues directed TDSC differentiation toward tenogenic, chondrogenic/fibrochondrogenic, and osteogenic lineages in the corresponding regions of the scaffold. In a rat patellar tendon–bone junction rupture model, implantation of the TDSC-seeded triphasic SF scaffold promoted superior regeneration and biomechanics.

Despite their promising tenogenic potential, TDSCs are a relatively new stem cell population in tissue engineering research and remain less extensively characterized compared to BM-MSCs and ADSCs [79]. Their low abundance (less than 5%) within tendon tissue and technical challenges associated with isolation and expansion continue to limit high-throughput investigation and clinical translation [80,81].

### 4.4. Tenocytes

Tenocytes are the principal resident cell type of tendon tissue, making up around 95%, and are responsible for maintaining ECM homeostasis and, consequently, tendon structure and function [82,83]. They exhibit limited proliferative and differentiation capacity compared with MSCs, so tenocyte-based approaches are primarily focused on the replenishment of native cells and restoration of ECM production within the tendon matrix. This concept is supported by preclinical in vivo studies illustrating that exogenous tenocytes can integrate into damaged or degenerated tendon tissue and contribute directly to matrix remodeling. In a rat Achilles tendon injury model, Güngörmüș et al. reported that implanted tenocyte-seeded decellularized tendon scaffolds promoted cellular repopulation, and in turn improved collagen organization and biomechanical properties [84]. Similarly, autologous tenocyte implantation (ATI) via injection into rabbit Achilles tendon injuries has been shown to enhance type I deposition, fiber alignment, and tensile strength [85].

ATI has now progressed to clinical application and is available in select centers in Australia and New Zealand. The procedure involves harvesting tenocytes from healthy tendon tissue, expanding them in culture, and reinjecting them into the injured tendon. Early reports suggest that ATI is safe, non-immunogenic, and promotes functional recovery [86]; for example, a 2013 case study described an elite gymnast returning to national-level competition following ATI treatment of a partial-thickness rotator cuff tear [87]. Additionally, numerous clinical studies in the past decade have utilized similar methods and have reported comparable clinical outcomes [86,87,88,89]. Clinical improvements include pain relief and progressive decreases in MRI T2 signal hyperintensity, indicative of reduced tissue damage, inflammation, and fluid accumulation [87,88,90]. Reported adverse effects appear minimal and rare, with transient injection site pain being the most common complication [89]. However, the current evidence base remains limited by small study populations and a lack of control groups, making it difficult to determine whether ATI directly accounts for observed clinical improvements [90]. Nevertheless, ATI represents a promising emerging approach for the treatment of chronic or treatment-resistant tendinopathies.

### 4.5. Induced Pluripotent Stem Cells (iPSCs)

iPSCs are patient-specific pluripotent cells generated through the reprogramming of adult somatic cells back into an embryonic-like state [91]. This process is achieved by introducing four transcription factors, known as the Yamanaka factors (OCT4, SOX2, KLF4, and c-MYC), which induce global epigenetic reprogramming and remodel chromatin structure [91,92]. iPSCs are capable of differentiating into most somatic cell types, including tenocytes, when exposed to appropriate developmental cues and growth factor signaling.

Komura et al. demonstrated that iPSC-derived tenocyte-like cells generated through a stepwise differentiation protocol exhibited elevated expression of tendon-related genes, including SCX, Mohawk, and TNMD [93]. When transplanted into a mouse Achilles tendon injury model, these cells reduced scar formation and improved collagen organization. Nakajima et al. generated iPSC tenocytes using a similar method, achieving 91.6% SCX-positive cells. Following transplantation into a rat Achilles tendon injury model, iPSC tenocytes promoted motor function recovery comparable to that of healthy tendon [94]. In both studies, these regenerative outcomes were mediated through a combination of direct engraftment and paracrine-driven enhancement of endogenous repair and matrix remodeling.

Besides stepwise growth factor signaling, genetic engineering represents another strategy for generating tenocyte-like iPSCs. Später et al. developed iTenocytes by overexpressing SCX iniPSC-derived MSCs (iMSCs), promoting a tenocyte-like phenotype. Nude rat Achilles tendon defects treated with SCX-overexpressing iMSCs seeded on an elastic collagen scaffold showed a well-organized connective tissue structure close to native tissue, as well as improved gait function [95].

Clinical translation remains limited due to concerns regarding genomic stability, potential tumorigenicity, and the need for strict differentiation protocols [96,97]. However, this approach is particularly important for older patients, as reprogramming reverses multiple hallmarks of cellular aging, including telomere shortening, mitochondrial dysfunction, and altered nuclear architecture, thereby improving the feasibility of autologous cell-based therapies. Their unlimited self-renewal provides a scalable cell source in cases where autologous tissue harvesting is limited, making iPSCs a potentially valuable, but technically demanding, option for tendon regeneration strategies [98,99].

### 4.6. Synovium-Derived Mesenchymal Stem Cells (Sy-MSCs or SM-MSCs)

Sy-MSCs are a population of multipotent stromal cells isolated from the synovial membrane of diarthrodial joints, typically the knee, and exhibit more robust proliferative and self-renewal properties than MSCs derived from bone marrow, periosteum, skeletal muscle, and adipose tissue [100]. These cells possess multilineage differentiation potential, including fibroblastic, chondrogenic, and osteogenic lineages [101]. Due to their strong propensity for fibrochondrogenic differentiation, they are particularly relevant for regeneration of fibrocartilage-rich regions, including tendon–bone junctions such as ligament entheses and rotator cuff attachments [100,102].

Ju et al.’s study established a rat patellar tendon-to-tibial bone tunnel model, mimicking an anterior cruciate ligament (ACL) reconstruction enthesis [103]. Sy-MSC treatment groups demonstrated accelerated tendon-to-bone healing, with significantly increased collagen fiber formation at the interface as early as 1 week, compared with controls. Dil labeling suggested some implanted cells localized to fibroblast-like regions, indicating possible fibroblast-like differentiation. However, despite these findings, studies on Sy-MSCs remain limited, and evidence for their use in mid-substance tendon repair is even more scarce. Table 2 summarizes key preclinical studies for the most commonly used cell types.

## 5. Clinical Trial Landscape

In addition to extensive preclinical studies, cell-based therapies have made considerable progress in clinical trials. Across completed and reported clinical trials listed on ClinicalTrials.gov, cell-based therapies have demonstrated a favorable safety profile, with no major treatment-related adverse events. Studies have evaluated both autologous [NCT01856140] and allogeneic [NCT02298023] cell therapies derived from adipose tissue and bone marrow [104,105]. In Lee et al.’s 2015 study, patients with lateral epicondylosis (LE) treated with ultrasound-guided allogeneic ADSC-fibrin experienced no significant adverse effects through 52 weeks of follow-up [106]. These findings were consistent across both dose groups (10^6^ and 10^7^ cells/mL), supporting the tolerability of ADSC administration at different concentrations [NCT01856140]. Similarly, Chun et al. (2022) reported no treatment-related adverse events following ADSC-fibrin injection for partial-thickness supraspinatus tendon tears (PTSRCCs) over a 2-year follow-up period [107]. Although transient pain was observed, it occurred in both cell and saline groups, suggesting that pain is likely associated with the injection procedure itself [NCT02298023]. Additional studies of autologous BM-MSCs for patellar tendinopathy (PT), Achilles tendinopathy (AT), and bone marrow aspirate concentrate (BMAC) for rotator cuff tears have also reported no serious adverse effects [104,105]. Overall, current evidence supports the tolerability of cell-based therapies for tendon repair in the short and intermediate term; however, larger studies with longer follow-up are required to further define their long-term safety.

With regard to efficacy, clinical trials have demonstrated promising, but variable, improvements in pain, function, and tendon structure following cell-based therapy. Among adipose-derived therapies, Lee et al. reported that ADSC-fibrin treatment for LE resulted in reduced elbow pain (VAS), improved elbow performance scores, and decreased ultrasound-detected structural tendon defects at 52 weeks [106]. However, no dose–response relationship was observed between the 10^6^ and 10^7^ cells/mL treatment groups, suggesting that higher cell concentrations may not provide additional clinical benefit. In a 2023 study, Lundeen et al. demonstrated improvements in total ASES score following uncultured autologous adipose-derived regenerative cell (UA-ADRC) treatment for partial-thickness rotator cuff tears (PTRCTs) [108] [NCT04077190]. Conversely, Chun et al.’s ADSC-fibrin study for PTSRCCs found no significant differences between cell-treated and control groups in activity-related pain, shoulder function, or tear size, highlighting the variability of outcomes among adipose-derived approaches [107].

Bone marrow-derived therapies, including BM-MSCs and BMAC, have also demonstrated potential clinical benefits, though outcomes vary across trials. Goldberg et al. (2024) reported improvements in pain and functional measures, including VAS, MOXFQ Pain, and VISA-A scores, at 24 weeks following autologous BM-MSC injection for AT [109] [NCT02064062]. However, the absence of a control group limits the interpretation of treatment-specific effects. Rodas et al. (2021) found that autologous BM-MSCs for PT significantly improved tendon structure at 6 months, while pain and functional scores (VAS and VISA-P) were not significantly different compared with the leukocyte-poor platelet-rich plasma (Lp-PRP) control group [104] [NCT03454737]. Centeno et al. (2020) evaluated BMAC for non-retracted supraspinatus tears and demonstrated improvements in pain, shoulder function, and tear size [105]. However, concurrent platelet product administration complicates whether these effects can be attributed to the cellular component alone [NCT01788683].

Overall, current evidence suggests that both adipose- and bone marrow-derived cell therapies may provide both clinical and structural benefits for tendon disorders. Nevertheless, variability in tendon pathology, treatment protocols, control groups, and outcome measures complicates direct comparison between studies and definitive evaluation of efficacy. In addition to completed trials, several ongoing, recruiting, or unpublished completed studies are evaluating various types of emerging cell-based approaches (Table 3). Results from these investigations may provide further insight into the safety, efficacy, and clinical applicability of these therapies across different tendon conditions. Larger randomized controlled trials with standardized protocols, appropriate controls, and longer follow-up are still needed to better define the role of cell-based therapies in tendon repair.

## 6. Regenerative/Repair Mechanisms

**Figure 1:**
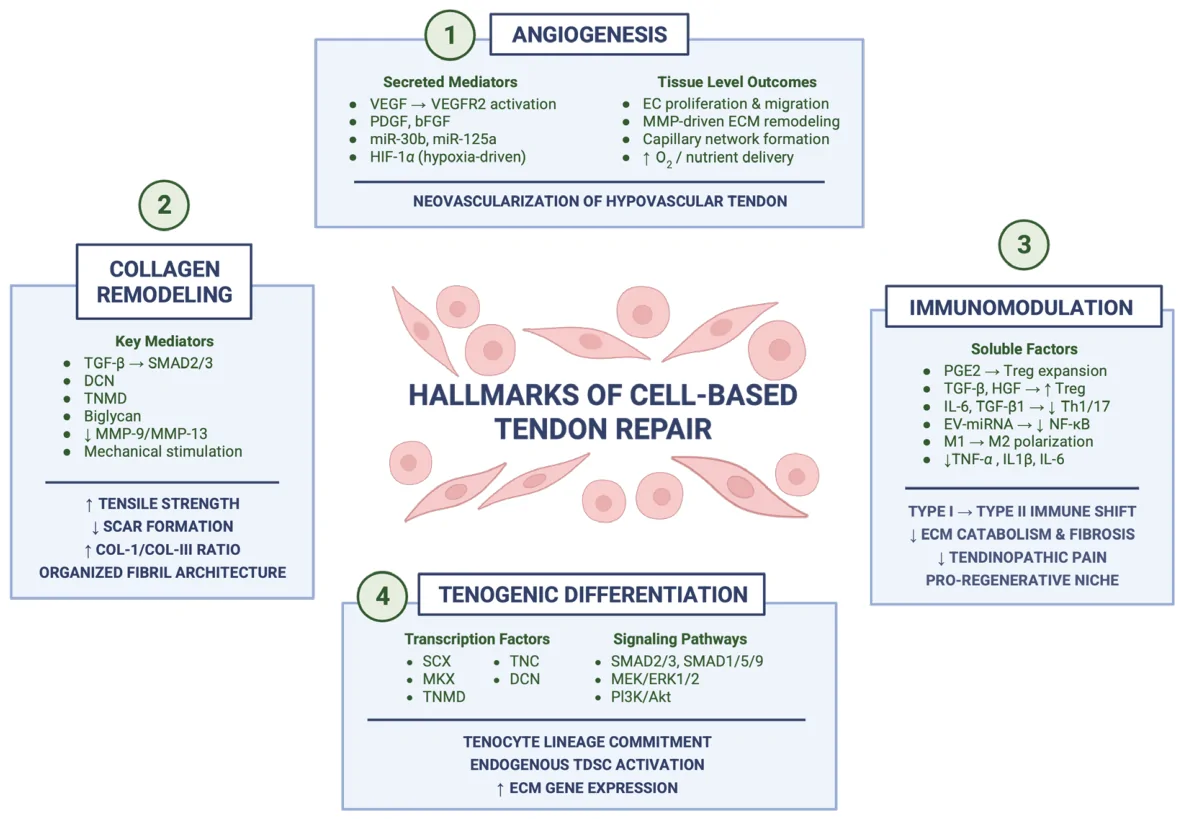
Hallmarks of cell-based tendon repair: key biological mechanisms driving functional tendon regeneration. Cell-based therapies promote tendon healing through four interconnected regenerative hallmarks: immunomodulation, angiogenesis, tenogenic differentiation, and collagen remodeling, orchestrated by paracrine mediators, extracellular vesicles, and cell–cell interactions. Together, these mechanisms attenuate inflammation, stimulate neovascularization and tenocyte lineage commitment, enhance extracellular matrix organization, and improve the structural and functional restoration of injured tendon tissue. The upward arrow indicates upregulation, and the downward arrow indicates downregulation.

Cell-based therapies promote tendon repair through a coordinated set of mechanisms, with paracrine signaling representing the primary mode of action of MSCs. These effects are mediated through growth factors, cytokines, and extracellular vesicles, which collectively regulate angiogenesis, immunomodulation, extracellular matrix remodeling, and tenogenic differentiation (Figure 1). Mechanical loading can further modulate these processes via mechanotransduction pathways.

### 6.1. Angiogenesis

Degenerative lesions typically occur in regions with poor vascularization, highlighting the importance of sufficient blood supply for tendon function. Following injury, angiogenesis becomes critical, as blood vessels must deliver oxygen, nutrients, and reparative cells to the site to aid in repair. MSCs boost this process primarily through secreting vascular endothelial growth factor (VEGF). Binding with its receptor, vascular endothelial growth factor receptor 2 (VEGFR2) directly enhances the proliferation and differentiation of vascular endothelial cells and induces the expression of matrix metalloproteinases (MMPs). These enzymes degrade the basement membrane and surrounding ECM components, clearing space for endothelial cells to migrate and establish new vascular networks [82]. Additional mediators, including platelet-derived growth factor (PDGF), basic fibroblast growth factor (bFGF), and pro-angiogenic microRNAs (miR30b and miR-125a), further support capillary development and branching in otherwise hypoxic tendon tissue [110].

Counterintuitively, hypoxia actually serves as a microenvironmental cue to initiate neovascularization, activating the hypoxia-inducible factor-1α (HIF-1α) pathway, and in turn upregulating pro-angiogenic genes [111]. Under normoxic conditions, HIF-1α is continuously synthesized, but rapidly degraded, through an oxygen-dependent mechanism. Prolyl hydroxylase domain proteins (PHDs) hydroxylate HIF-1α, allowing recognition by the von Hippel–Lindau (VHL) E3 ubiquitin ligase complex, which targets HIF-1α for proteasomal degradation. Reduced oxygen availability limits PHD activity, preventing HIF-1α hydroxylation and subsequent degradation [112]. HIF-1α then accumulates in the cytoplasm, translocates into the nucleus, and complexes with HIF-1β to form an active HIF-1 transcriptional complex. This complex binds to hypoxia-responsive elements (HREs) within target gene promoters, driving the transcription of genes involved in angiogenesis, metabolism, and ECM remodeling [113].

A major downstream effect of HIF-1ɑ activation is the upregulation of VEGF and other angiogenic factors, including PDGF, bFGF, and angiopoietins [114]. These factors collectively promote endothelial recruitment, vessel maturation/stabilization, and pericyte activity. Therefore, combining controlled hypoxic stimulation with cell-based therapies may further enhance paracrine angiogenic activity. Hypoxia preconditioning of MSCs has shown to improve their regenerative capacity, with hypoxia-preconditioned rat MSCs demonstrating better healing potential in Achilles tendon injury models [115,116].

Overall, enhanced angiogenesis improves the delivery of oxygen, nutrients, and reparative cells to the otherwise hypovascular tendon environment, supporting early healing and regeneration responses.

### 6.2. Collagen Synthesis

In parallel, cell-based therapies play an important role in regulating the synthesis and organization of collagen—the main component of tendon. Following injury, fibroblasts initially deposit type III collagen to stabilize the wound [117]. This immature matrix is subsequently replaced with type I collagen, which provides the organized fibrillar architecture and high tensile strength characteristic of healthy tendon tissue. In chronic tendinopathy, this transition from type III to type I collagen is often impaired, resulting in persistent accumulation of disorganized, fibrotic, type III collagen-rich tissue that lacks the mechanical properties required for normal tendon function.

Among the numerous signaling pathways involved in tendon repair, transforming growth factor-beta (TGF-β) is considered a key regulator of ECM synthesis and remodeling. TGF-β is released endogenously following tendon injury [118], and MSCs can boost its abundance through paracrine secretion [119]. In its canonical pathway, TGF-β binds to type I and II serine/threonine kinase receptors on fibroblasts and tenocytes, inducing phosphorylation of SMAD2/3. Phosphorylated SMAD2/3 (p-SMAD2/3) forms a complex with SMAD4 and translocates to the nucleus, where it regulates the transcription of collagen-related genes, including COL1A1, COL1A2, COL3A1, as well as other ECM proteins [120,121]. TGF-β not only stimulates collagen production by existing tenocytes, but also expands the pool of collagen-producing cells by promoting the recruitment and differentiation of surrounding progenitor cells [121,122].

Numerous studies have demonstrated that increased TGF-β/SMAD signaling promotes collagen synthesis [123], whereas inhibition or deletion of this pathway reduces collagen production and delays tendon healing [124]. However, excessive or prolonged TGF- β signaling may contribute to fibrosis, scar formation, and peritendinous adhesions, highlighting the importance of maintaining a balance between matrix deposition and remodeling [125]. Supporting this mechanism, ADSC-derived exosomes increased p-SMAD2/3 levels, promoting TSC proliferation, migration, and tenogenic differentiation while enhancing collagen I expression and collagen fiber organization during tendon healing in a rat model [126]. These effects were minimized with the addition of SMAD2/3 inhibitor SB431542, confirming that regenerative responses were mediated through this pathway.

Additional ECM proteins secreted by exogenous cell therapies, including decorin (DCN), tenomodulin (TNMD), and biglycan, regulate collagen maturation and organization. DCN controls collagen fibril diameter and spacing, TNMD promotes tendon migration as well as tenocyte proliferation and migration, while biglycan modulates collagen organization, matrix maturation, and tenocyte migration [127]. In a rabbit Achilles tendon repair model, treatment with ADSC-derived EVs increased the expression of collagen I, DCN, TNMD, and biglycan, while reducing collagen III expression compared with controls [127]. These changes were accompanied by improved collagen fiber alignment and more mature tendon architecture, suggesting a shift away from persistent fibrotic, collagen III-rich scar tissue toward organized collagen I-rich tendon. Hypoxia preconditioning of MSCs further enhanced the expression of collagen I, DCN, and TNMD, producing histological features that most closely resembled normal uninjured tendon, including improved collagen organization on H&E and Masson’s trichrome staining and stronger collagen I and TNMD immunohistochemical staining [128].

Mechanical loading further augments collagen synthesis through mechanotransduction, the process by which cells convert physical forces into biochemical signals [129]. Physiological tensile stimulation activates mechanosensitive pathways, including integrin-associated focal adhesion kinase (FAK) signaling, which enhances TGF-β signaling and increases SMAD2/3 phosphorylation [130]. In vivo studies have demonstrated that mechanical forces promote TGF-β-dependent SMAD activation in tenocytes, contributing to ECM organization and tendon adaptation to loading [131]. In another study, Zhang et al. demonstrated that combining BMSC therapy with cyclic mechanical stimulation and TGF-β1 enhanced tenogenic differentiation, increased mechanical properties, and improved tendon regeneration in a rat Achilles tendon repair model [132]. Thus, integrating cell-based therapies with controlled mechanical loading may amplify the regenerative effects of paracrine signaling by promoting collagen synthesis, fiber alignment, and maturation of a collagen I-rich ECM.

Collectively, these mechanisms shift the balance towards organized, type I collagen, supporting the restoration of tendon structure and biomechanical function. This shift is critical for restoring the structural integrity of the tendon, and for improving tensile strength during the remodeling process.

### 6.3. Immunomodulation

Besides angiogenesis and collagen synthesis, immunomodulation is a key mechanism through which cell-based therapies aid in tendon repair. Following tendon injury, immune activation occurs through a coordinated balance between type I and type II immune responses. The type I response is characterized by activation of pro-inflammatory pathways involving M1-like macrophages, Th1, and Th17 cells, which produce cytokines such as IL-1β, TNF-α, and IFN-γ to promote immune cell recruitment and matrix degradation [133]. In contrast, the type II response promotes inflammation resolution and repair via M2-like macrophages, regulatory T cells (Tregs), and anti-inflammatory mediators, such as IL-10 and TGF-β [134]. The balance between these two responses is important, as persistent type I activity, or uncontrolled inflammation, can drive excessive ECM degradation, scar formation, impaired healing, and chronic pain [135].

Cell-based therapies promote a shift towards type II immune responses by enhancing reparative immune cell phenotypes while suppressing excessive inflammatory activity. MSCs mediate these effects by secreting soluble immunoregulatory factors such as PGE2, TGF-β, and hepatocyte growth factor (HGF), which induce proliferation and activation of Tregs to maintain immune homeostasis [136]. Simultaneously, their release of TGF-β1, IL-6, and PGE2 inhibits the proliferation of Th1 and Th17 cells and M1-type macrophages, reducing pro-inflammatory cytokine production [137].

MSC-derived extracellular vesicles (MSC-EVs), including MSC-Exos, further amplify these immunomodulatory effects by delivering regulatory microRNAs and suppressing NF-κB signaling pathways. These mechanisms reduce expression of M1 macrophage markers, promoting a more reparative macrophage phenotype within injured tendon tissue [138]. These immunomodulatory pathways have been linked to improved pain-related behaviors in preclinical animal models, and reduced severity of tendinopathic symptoms in clinical studies [139]. By limiting excessive catabolic activity, nociceptive sensitivity, and inflammatory signaling, MSC-based therapies establish a more balanced immune microenvironment that is more conducive to tendon remodeling and regeneration.

### 6.4. Tenogenic Differentiation

Ultimately, complete restoration of tendon structure requires the proliferation and migration of surrounding progenitor cells, and their differentiation into functional tenocytes. In native tendon, these processes are inherently slow due to the hypovascular and hypocellular environment. Cell-based therapies overcome these limitations by delivering mature tenocytes or progenitor cells capable of tenogenic differentiation, while also stimulating resident cells to adopt a tenogenic fate.

Among stem cell populations, TDSCs are most easily induced into tenocytes, likely reflecting their native tissue origin. Nevertheless, other cell types have also demonstrated the capacity to promote tendon regeneration. For example, BM-MSCs induced a greater expression of tendon-associated genes, including type I collagen, DCN, and tenascin, than ADSCs in an equine flexor tendon model, consistent with superior histological repair [120]. Alternatively, the use of fully differentiated tenocytes avoids the risk of off-target differentiation, although they lack the angiogenic and immunomodulatory properties of stem cell-based therapies.

Successful tenocyte differentiation is guided by the presence of growth factors (e.g., bFGF, TGF-β1) that include the expression of tendon-specific markers such as scleraxis (SCX), TNMD, Mohawk (MKX), tenascin-C (TNC), type I collagen, and DCN [82]. Beyond its established role in collagen synthesis, as discussed previously, TGF-β/SMAD signaling also contributes to tenocyte lineage specification. MSC-Exos deliver bioactive cargo that activate SMAD2/3 and SMAD1/5/9 signaling pathways, increasing expression of tenogenic markers including SCX, TNMD, and collagen I [126]. In parallel, MSCs promote tenocyte proliferation, migration, and survival through activation of MEK/ERK1/2 and PI3K/Akt signaling pathways [126,140].

These signaling mechanisms have been incorporated into co-culture and stepwise differentiation protocols that direct injected MSCs and iPSCs toward a tenogenic lineage. Using a SCX-EGFP tendon-specific reporter system, Komura et al. developed a stepwise protocol, mimicking embryonic tendon development and differentiation through the early mesendoderm, mesoderm, etc. [93]. These protocols may expand the tenogenic potential of otherwise less committed cell populations, helping replenish tendon-like cells at sites of injury or degeneration, and ultimately supporting both structural and functional restoration of tendon tissue.

In addition to biochemical regulation, mechanical stimulation plays a critical role in regulating tenocyte fate and behavior through mechanotransduction pathways. As tendons are highly mechanosensitive tissues, physiological loading is essential for maintaining tenocyte phenotype, ECM homeostasis, and collagen fiber alignment [141]. Appropriate mechanical stimulation enhances tendon-specific expression, including SCX and MKX, to promote tenogenic differentiation and maturation of progenitor cells [142]. In contrast, excessive loading or stress deprivation disrupts matrix homeostasis and promotes degenerative remodeling or adipogenic, chondrogenic, and osteogenic lineages [143]. Therefore, the success of cell-based therapies depends not only on the intrinsic tenogenic potential of transplanted cells, but also on exposure to an appropriate biochemical and mechanical environment that supports tenogenic differentiation, cellular maturation, and functional ECM formation.

## 7. Limitations

Despite their promise, cell-based therapies for tendon repair face significant limitations, especially in translating in vitro findings into in vivo applications. Still, injected or transplanted cells show inadequate survival and integration into tendon tissue, leading to a better, but limited, range of motion and biomechanical function. Both allogeneic and autologous cells still carry risks of infection, pathogenic transmission, and unfavorable inflammatory reactions. Progenitor cell therapies, especially those involving pluripotent populations, raise concerns of misdifferentiation, which can lead to the formation of ectopic tissue and teratomas. Additionally, long-term safety data are limited, and practical challenges such as cost, accessibility, and scalability should be considered, especially as existing treatments do not address these concerns. Furthermore, standardization remains a major challenge with variability in cell source, dose, timing, administration, and assessment technique. This is further complicated by patient-specific factors such as age, metabolic health, immune status, and the severity or location of the injury. For example, a standardized treatment may cause greater pain in a patient with an Achilles tendon injury, a site more susceptible to long-term inflammation, than one with a rotator cuff injury. Such heterogeneity complicates efforts at standardization, but at the same time creates an opening for personalized cell-based therapeutics.

## 8. Future Directions

Despite the promising potential of cell-based approaches in tendon and soft-tissue repair, significant advancements remain to be made. Future studies should focus on establishing evidence-based criteria for selecting the optimal cell therapy according to patient profile and tendon pathology. Prospective studies should further characterize the regenerative potential of cell subtypes, including BM-MSCs, ADSCs, TDSCs, IPSC-derived cells, and tenocytes, across patients with differing age, body mass index (BMI), metabolic status, and disease characteristics. Systematic screening of these cell populations using functional assays and molecular profiling may help identify which cell sources are best suited for which clinical scenarios. Such studies could determine whether specific patient populations or injury patterns consistently benefit from a specific cell source. For instance, ADSCs may be advantageous in patients requiring rapid cell expansion, while iPSC-derived cells may better overcome age-related senescence.

The choice between autologous and allogeneic cell sources is also a critical consideration for patient-specific therapy. By using the patient’s own cells, autologous cell therapies offer excellent biocompatibility with little to no risk of immune rejection or disease transmission [144]. However, their use is limited by donor-site morbidity, additional and/or invasive surgical procedures, prolonged manufacturing times, and reduced cell quality in older patients or those with comorbidities [145]. In contrast, allogeneic sources provide a standard, abundant supply of cells with immediate availability and consistent quality control, making them particularly attractive when autologous tissue is limited or rapid treatment is required. Although MSCs possess immunomodulatory properties [146], allogeneic therapies still carry the risk of host immune responses, graft rejection, and HLA incompatibility [50]. Given the advantages and limitations of both approaches, future studies should establish standardized screening strategies to (1) assess the quality and regenerative potential of autologous cells and (2) streamline allogeneic donor selection and HLA compatibility assessment.

Another important direction concerns translating molecular biomarkers into clinically useful assays for cell-based therapies. As discussed, key molecular pathways, including HIF-1α, TGF-β/SMAD, NF-κB, and PI3K/Akt, along with tenogenic markers, such as SCX, TNMD, and MKX, have been implicated in angiogenesis, immunomodulation, ECM remodeling, and tenogenic differentiation. Future comparative transcriptomic, proteomic, and secretome analyses could identify biomarker signatures associated with regenerative potential, improving prediction of therapeutic efficacy and enabling development of standardized potency assays. These biomarkers may subsequently guide optimization strategies, including hypoxic conditioning, co-culture approaches, or targeted genetic modification. Co-culture systems could be designed to promote favorable cell–cell interactions and enrich therapeutic cells with desired EV or EXO cargo. Similarly, genetic modification may be used to enhance the expression of key growth factors and key pathway modulators to enhance reparative efficacy. Such modifications may improve cell retention, proliferation, or tendon phenotype. Supporting this approach, two studies using SCX-transduced MSCs demonstrated improved repair outcomes: Gulotta et al. reported that SCX-BM-MSCs increased ultimate stress-to-failure and stiffness scores, while Tan et al. found that SCX-TDSCs improved both histological organization and biomechanical outcomes [63,76]. However, genetic modification introduces additional safety concerns, including off-target effects, genomic instability, and tumorigenicity, that need to be carefully evaluated before clinical translation. Comparative studies evaluating unmodified, co-culture-primed, and genetically engineered cell populations will be necessary to determine the most effective and safest strategies for specific tendon pathologies.

Beyond optimizing cellular and molecular characteristics, future work should also focus on developing scaffolds that more closely resemble the structure, composition, and mechanical properties of native tendons. Although current collagen, decellularized ECM, and silk scaffolds do attend to needs of structural support and cell delivery, further refinement is required to address the unique spatial, mechanical, and biochemical demands of different injury sites. To tackle this, modern scaffolds may incorporate adjustable mechanical properties, controlled degradation profiles, and region-specific biochemical cues. Future studies should investigate how scaffold designs interact with different cell sources and at different injury sites to determine the most effective combinations for specific tendon pathologies. Recent advances, like Geng et al.’s triphasic (collagen I, TGF-β1, and HA) silk-fibrin scaffolds that recreate native tendon–bone compositional gradients, may provide a promising framework for more precise regenerative therapies [78]. An emerging scaffold-free method, cell sheet implantation, represents an alternative approach to enhance cell delivery, retention, and therapeutic efficacy. Compared with traditional cell injection, cell sheets preserve endogenous ECM and cell–cell interactions, while improving attachment to and viability at the target tissue [147]. However, their reliance on a minor surgical procedure may limit clinical practicality. To find a balance between regenerative potential, invasiveness, and clinical feasibility, comparative studies evaluating cell injection, scaffold-based systems, and cell sheet approaches will be necessary.

Despite the potential for precision regenerative therapies, successful clinical implementation will require rigorous standardization. Future studies should establish consistent protocols for cell isolation, expansion, characterization, dosing, and delivery. Defining reproducible potency assays, screening workflows, and clinically relevant outcome measures will be essential for comparing therapeutic approaches and consistency across studies. Together, these strategies may enable the development of more reliable and clinically translatable cell-based therapies for tendon repair.

## Figures and Tables

**Table 1:** Comparative summary of cell types for tendon repair.

Cell Type	Source	Tenogenic Potential	Expansion Capacity	Paracrine Capacity	Advantages	Limitations	Clinical Translation
**BM-MSCs**	Bone marrow	Moderate–high	Moderate	High	Most extensively studied; strong immunomodulatory and regenerative effects	Invasive harvest procedure; low cell yield; donor age affects potency	Advanced preclinical studies, early clinical applications
**ADSCs**	Adipose tissue	Moderate	High	High	Abundant source; minimally invasive harvest; rapid proliferation; strong paracrine activity	Slightly lower tenogenic commitment than BM-MSCs; donor variability	Advanced preclinical studies, early clinical applications
**TDSCs**	Tendon tissue	Very high	Moderate	Moderate	Intrinsically tendon-committed; superior expression of tendon markers; strong tendon-specific repair potential	Difficult isolation; low abundance	Preclinical
**Synovial MSCs**	Synovial membrane	Moderate	High	High	Robust proliferation; potential for tendon–bone interface regeneration	Limited evidence in tendon repair; less tendon-specific than TDSCs	Preclinical
**iPSCs**	Somatic cells	Very high	Very high	Moderate	Unlimited expansion; patient-specific; rejuvenated cellular phenotype; strong differentiation potential	Tumorigenicity concerns; genomic instability; complex manufacturing	Early preclinical
**Tenocytes**	Tendon tissue	Already differentiated	Low	Low	Native tendon cell phenotype; direct ECM synthesis; no risk of stem cell misdifferentiation	Limited proliferative capacity; donor-site morbidity; difficult expansion	Limited clinical use (ATI)

**Table 2:** Summary of key preclinical studies evaluating cell-based therapies for tendon repair.

Study	Cell Type	Study Type	Model	Delivery Method
Crovace et al. [57]	BM-MSCs	In vivo	Ovine Achilles tendinitis	Intralesional injection
Smith et al. [58]	BM-MSCs	In vivo	Equine superficial digital flexor tendon (SDFT) injury	Intratendinous injection with bone marrow carrier
Yokoya et al. [59]	BM-MSCs	In vivo	Rabbit infraspinatus tendon tear	Cell-seeded PGA scaffold
Zhao et al. [60]	BM-MSCs	Ex vivo	Canine flexor digirum profundus tendon laceration	Cell-seeded collagen gel patch at lacerated ends
Smith et al. [61]	BM-MSCs + rehabilitation	In vivo	Equine SDFT lesion	Intralesional ultrasound-guided injection combined with rehabilitation
Flück et al. [62]	BM-MSCs	In vivo	Ovine rotator cuff tear (infraspinatus release)	Electropulse-assisted intramuscular (infraspinatus) injection
Gulotta et al. [63]	BM-MSCs (SCX-transduced)	In vivo	Rat rotator cuff injury	Intratendinous injection with fibrin glue carrier
Norelli et al. [65]	ADSCs (GDF-5,6,7 and PDGF-BB-conditioned)	In vivo	Rat Achilles tendon injury	Hydrogel-based cell delivery
de Lima Santos et al. [66]	ADSCs + suture	In vivo	Rabbit flexor digitorum superficialis tendon lesion	Intralesional injection + suture
Shin et al. [67]	ADSCs	In vivo	Rat rotator cuff tear	RCT repair + cell sheet implantation
Veronesi et al. [68]	ADSCs (OVX)	In vitro	OVX tenocyte co-culture	Indirect co-culture (ADSCs + tenocytes)
Ahrberg et al. [69]	ADSCs	In vivo	Equine SDFT tendinopathy	Intralesional injection
Al-Ani et al. [73]	TDSCs	In vivo	Rat Achilles tendon rupture	Intratendinous injection
Khaled et al. [74]	TD-MSCs	In vivo	Rat model of Achilles tendon injury	Intratendinous injection
Lui et al. [75]	TDSCs (CTGF/ascorbic acid-conditioned)	In vitro and in vivo	Rat patellar tendon window injury	Fibrin-based cell construct transplantation
Tan et al. [76]	TDSCs (Scx-transduced)	In vivo	Rat patellar tendon window injury	Fibrin-based cell construct transplantation
Wen et al. [77]	TDSCs	In vivo	Rabbit rotator cuff injury	Cell-seeded acellular porcine Achilles tendon (APAT) patches
Geng et al. [78]	TDSCs	In vivo and in vitro	Rat patellar tendon–bone junction rupture	Cell-seeded triphasic (collagen I, TGF-B1, HA) silk-fibrin scaffold
Güngörmüş et al. [84]	Tenocytes	In vivo	Rat Achilles tendon injury	Cell-seeded decellularized tendon matrices
Chen et al. [85]	Tenocytes	In vivo	Rabbit Achilles tendon injury	Intralesional injection
Wang et al. [86]	Tenocytes	Clinical	Human chronic lateral epicondylitis	Ultrasound-guided ATI
Wang et al. [87]	Tenocytes	Clinical	Human partial-thickness rotator cuff tear	Ultrasound-guided ATI
Bucher et al. [89]	Tenocytes	Clinical	Human chronic recalcitrant gluteal tendinopathy	Ultrasound-guided ATI
Schwab et al. [88]	Tenocytes	Clinical	Human subscapularis rotator cuff injury	Ultrasound-guided ATI
Komura et al. [93]	iPSC-derived tenocytes	In vivo	Mouse Achilles tendon injury	Cell-collagen construct transplantation
Nakajima et al. [94]	iPSC-derived tenocytes	In vivo	Rat Achilles tendon injury	Intralesional injection with hydrogel matrix
Später et al. [95]	iPSC-derived MSCs (Scx-overexpressing)	In vivo	Nude rat Achilles tendon defect	Cell-seeded elastic collagen scaffold
Ju et al. [103]	Synovial MSCs	In vivo	Rat tendon-to bone tunnel	DiI-labeled cell transplantation

**Table 3:** Summary of clinical trial evaluating cell-based therapies for tendon repair.

ClinicalTrials.gov ID	Intervention	Condition	Study Start	Study Completion	Phase	Status	Responsible Party
NCT01687777	Arthroscopic or mini-open rotator cuff repair + autologous MSCs (collagen I membrane)	Rotator cuff tear (supraspinatus)	2010-05	*2013-12 (Est.)*	Phase II	Unknown status	Hospital San Carlos, Madrid
NCT01343836	Autologous tenocytes + eccentric exercise	Chronic Achilles tendinopathy	2011-04	2014-06	Phase II/III	Completed	Erasmus Medical Center
NCT03068988	Open rotator cuff repair + concentrated BM-MSCs	Rotator cuff tear (supraspinatus)	2012-01-01	*2021-12-31 (Est.)*	Phase I	Unknown status	Hospital Znojmo
NCT01788683	Autologous BMAC	Rotator cuff tear (supraspinatus)	2013-02	2022-07-28	-	Completed	Regenexx, LLC
NCT01856140	Allogeneic ASCs	Lateral epicondylitis	2013-05	2018-04	Early Phase I	Completed	Seoul National University Hospital
NCT02131077	Allogeneic ADSCs	Lateral epicondylitis	2014-01	2015-08	Phase I/II	Completed	Anterogen Co., Ltd.
NCT02298023	Allogeneic ADSCs (fibrin)	Partial-thickness rotator cuff tear (supraspinatus)	2014-09	2018-04-10	Phase II	Completed	Seoul National University Hospital
NCT02064062	Autologous BM-MSCs	Achilles tendinopathy	2015-01	-	Phase II	Unknown status	University College, London
NCT02484950	Arthroscopic rotator cuff repair + autologous BM-MSCs	Full-thickness rotator cuff tear	2015-11-15	2024-06-27	-	Completed	Rush University Medical Center
NCT02918136	Autologous ADRCs	Partial-thickness rotator cuff tear	2016-12	2018-12-13	-	Completed	InGeneron, Inc.
NCT03454737	Autologous BM-MSV	Patellar tendinopathy	2017-12-13	2020-12-14	-	Unknown status	Institut de Terapia Regenerativa Tissular
NCT03449082	Allogeneic ASCs (fibrin)	Intractable common extensor tendon injury	2018-03-15	2021-03-08	Phase II	Completed	Seoul National University Hospital
NCT03279796	Autologous ADSCs (x3)	Rotator cuff tear or lateral epicondylitis	*2018-10-01 (Est.)*	*2021-12-31 (Est.)*	Phase II	Unknown status	Anterogen Co., Ltd.
NCT03752827	Autologous ADRCs	Partial-thickness rotator cuff tear	2019-05-13	*2025-12 (Est.)*	-	Active, not recruiting	InGeneron, Inc.
NCT03332238	Rotator cuff repair + SVF (Ringer’s solution)	Full-thickness rotator cuff tear (supraspinatus)	2019-07-01	*2026-07-23 (Est.)*	Phase II	Active, not recruiting	Hospital for Special Surgery, New York
NCT04077190	Autologous ADRCs	Partial-thickness rotator cuff tear	2019-08-01	2021-12-01	-	Completed	InGeneron, Inc.
NCT04670302	Open rotator cuff repair + allogeneic AAdMSC-HAM composite	Rotator cuff tear (supraspinatus)	2019-10-17	2022-12-31	-	Unknown status	Dr. Soetomo General Hospital
NCT03688308	Arthroscopic rotator cuff repair + autologous BMAC	Rotator cuff tear	*2020-01-01 (Est.)*	*2021-10 (Est.)*	-	Withdrawn	Stanford University
NCT03362424	Arthroscopic rotator cuff repair + MSCs	Full-thickness rotator cuff tear (posterosuperior)	2020-11-01	*2023-12-01 (Est.)*	Phase II	Suspended	University of Sao Paulo
NCT06505135	Arthroscopic rotator cuff repair + autologous MFAT	Rotator cuff tear (supraspinatus)	2024-09-03	*2026-09 (Est.)*	Phase I	Enrolling by invitation	University of Southern Denmark
NCT06361797	Arthroscopic rotator cuff repair + autologous BMC	Rotator cuff tear	*2025-02 (Est.)*	*2027-12 (Est.)*	-	Withdrawn	Johns Hopkins University
NCT07320378	Arthroscopic rotator cuff repair + autologous TSPCs (fibrin/thrombin scaffold)	Rotator cuff tendinopathy	*2026-05 (Est.)*	*2029-01 (Est.)*	Early Phase I	Not yet recruiting	Second Affiliated Hospital, School of Medicine, Zhejiang University
NCT07592936	Arthroscopic rotator cuff repair + autologous MFAT (x3)	Rotator cuff tear (supraspinatus)	*2026-08 (Est.)*	*2028-08 (Est.)*	Phase I/II	Not yet recruiting	University of Southern Denmark

Abbreviations: MSC, mesenchymal stem cell; BM-MSC, bone marrow-derived mesenchymal stem cell; BMAC, bone marrow aspirate concentrate; PRP, platelet-rich plasma; PL, plasma lysate; BMC, bone marrow concentrate; ADSC, adipose-derived stem cell; ADRC, adipose-derived regenerative cell; SVF, stromal vascular fraction; MFAT, microfragmented adipose tissue; AAdMSC, allogeneic adipose-derived mesenchymal stem cell; HAM, human amniotic membrane; TSPC, tendon stem/progenitor cell; MSV, mesenchymal stromal vascular product; RCT, rotator cuff tear; Est., estimated; x3, three administrations/doses.

## Data Availability

No new data were created or analyzed in this study. Data sharing is not applicable to this article.
